# Supplementary material for: Wnt/β-Catenin-Pathway Alterations and Homologous Recombination Deficiency in Cholangiocarcinoma Cell Lines and Clinical Samples: Towards Specific Vulnerabilities
Source: J Pers Med. 2022 Aug 1;12(8):1270. doi: 10.3390/jpm12081270 (PMC9410222; doi:10.3390/jpm12081270)
Supplement: Supplementary file 1 [file jpm-12-01270-s001.zip › jpm-1799777-supplementary.pdf]

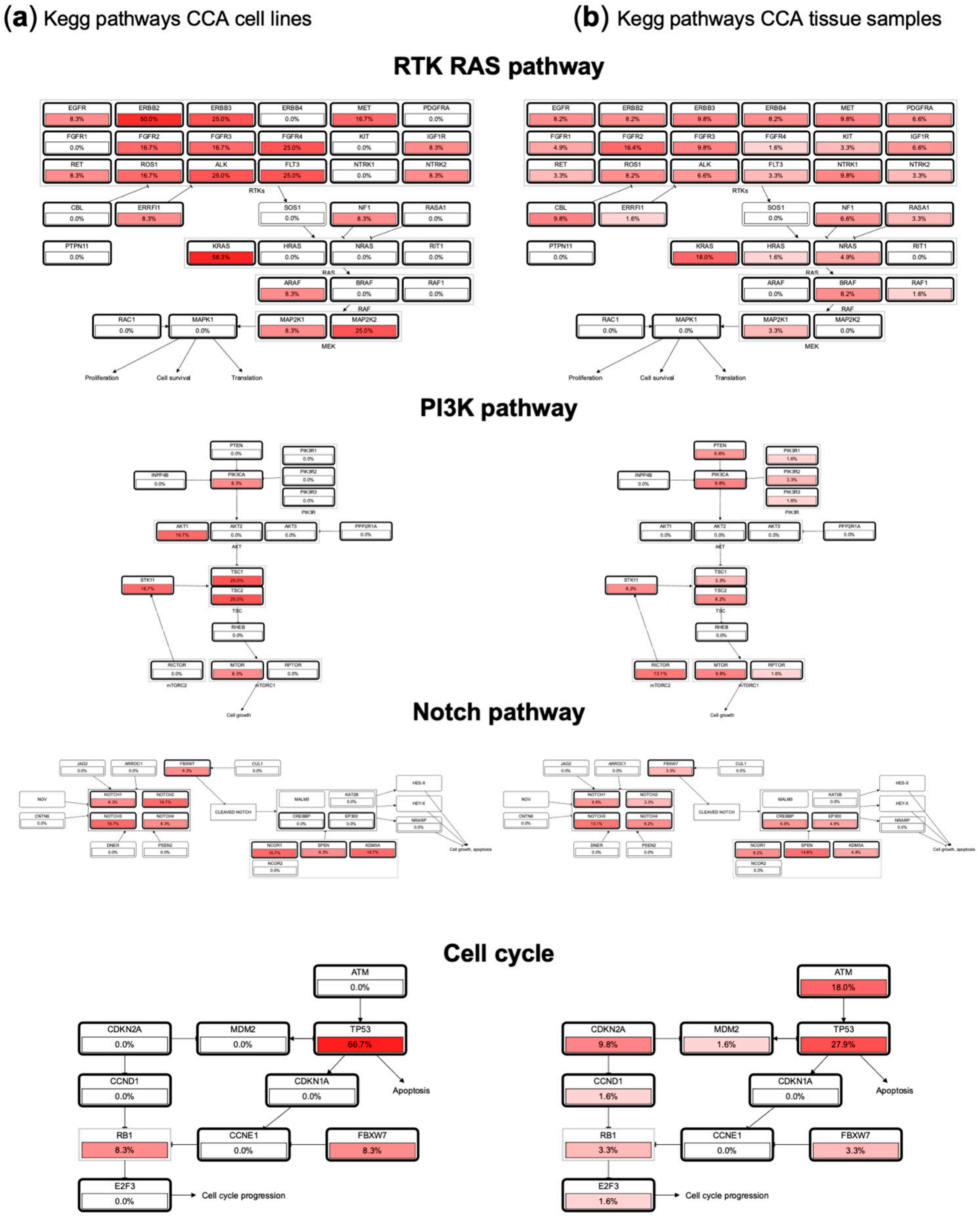

Figure S1. (**a**) Maps of different Kegg pathways with alterations in CCA cell lines. The mutational frequencies, including putative driver mutations and variants of unknown significance, are given. (**b**) Mutational frequencies in CCA tissue samples.

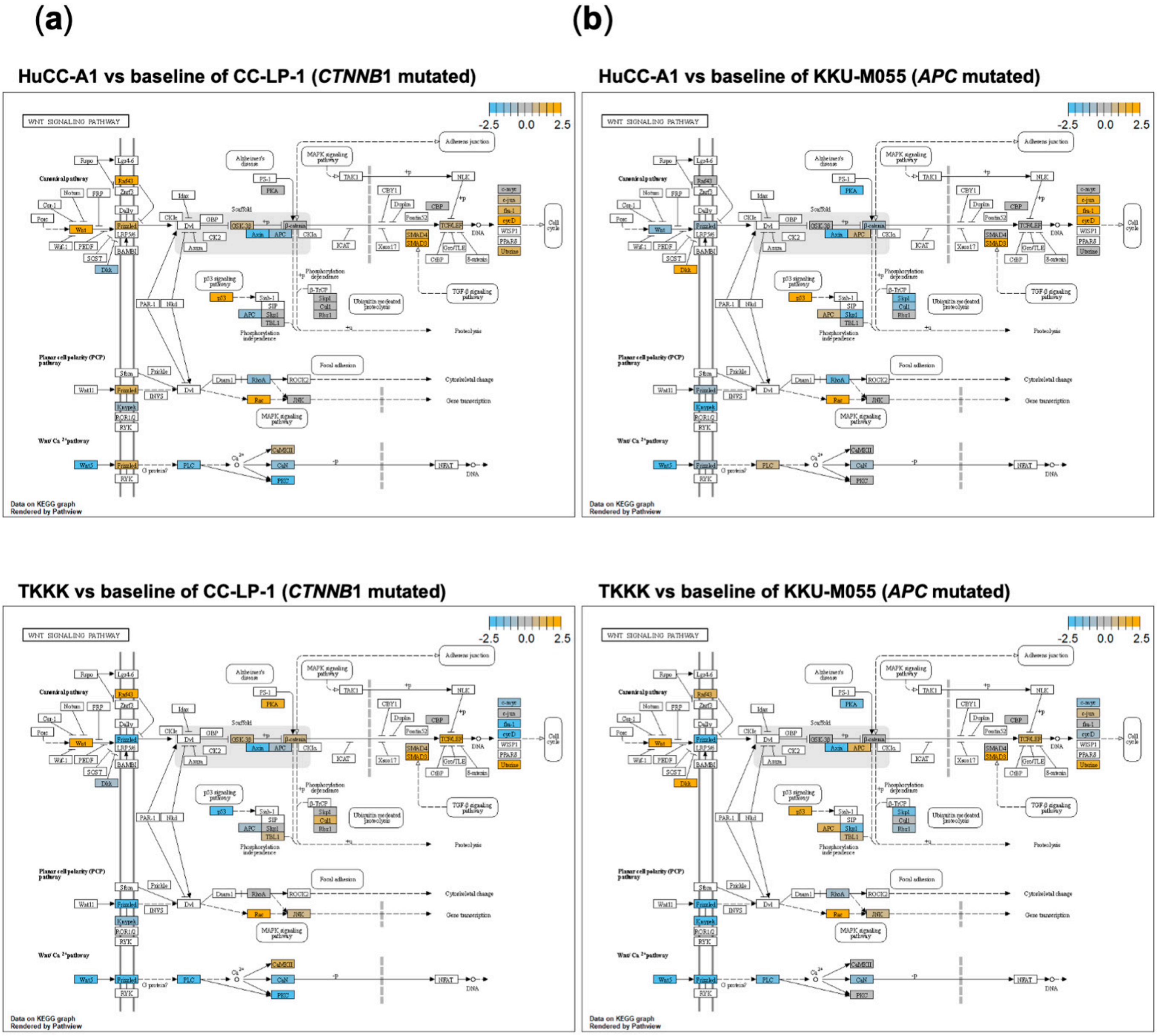

Figure S2. Differential expression of Kegg WNT-pathway annotated genes (as determined by Nanostring®) as rendered by Pathview. Orange indicates high scores; blue indicates low scores (log2-fold-change). Genes are displayed irrespective of statistical significance, i.e., fold-change is a mean value of three replicate measurements. (**a**) Wild type cell lines against the baseline of *CTNNB1*-mutated CC-LP-1 and (**b**) against the baseline of *APC*-mutated KKU-M055 cell lines.

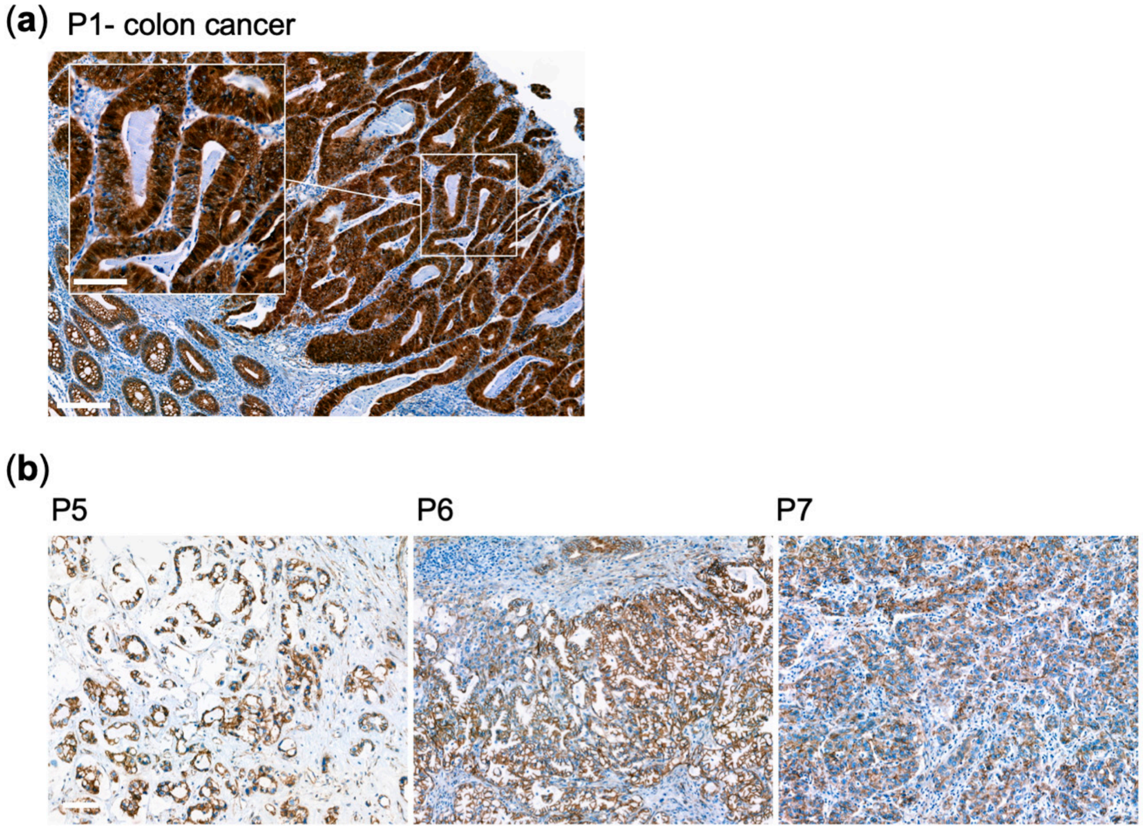

Figure S3. (**a**) β-catenin immunohistochemistry of metachronous colon cancer in P1. (**b**) β-catenin immunohistochemistry of WNT-pathway wildtype CCA samples (P5 – P7). Scale bar in (a) 200 μm, inset 50 μm, in (b) 100 μm P, patient identifier.

Table S1. OncoKB driver mutations cell lines overview.

	KRAS	TP53	SMAD4	SMARCA4	IDH1	ARID1A	ARID2	RBM10	RAD51D	TERT	KMT2A	DNMT3A	MAP2K1	APC	NSD1	CTNNB1	EPHA3	TSC1	FBXW7	SETD2	SPEN
**YSCCC**	WT	V272M	WT	E457*	R100Q	WT	WT	WT	K111Ifs*13	Promoter	S82*	WT	WT	WT	E531Q	WT	WT	X36_splice	WT	WT	WT
**TKKK**	WT	Q104*	WT	WT	WT	WT	K569Nfs*12	WT	WT	WT	WT	WT	WT	WT	WT	WT	WT	WT	WT	WT	WT
**RBE**	G12V	WT	WT	WT	R132S	WT	WT	WT	WT	WT	WT	WT	WT	WT	WT	WT	WT	WT	WT	WT	WT
**OZ**	Q61L	WT	WT	WT	WT	WT	WT	WT	WT	WT	G3131S	WT	WT	WT	WT	WT	WT	T899S	WT	T2421A	WT
**KKU-M213**	G13C	V31I	R361C	WT	WT	G324V	WT	E748*	WT	WT	WT	WT	WT	WT	X1308_splice	WT	WT	WT	WT	WT	WT
**KKU-M156**	G13C	V31I	R361C	WT	WT	G92A	WT	E748*	WT	WT	WT	WT	WT	WT	WT	WT	WT	F503L	WT	WT	WT
**KKU-100**	G12D	WT	X380_splice	K1412*	WT	WT	WT	WT	WT	WT	WT	WT	WT	WT	WT	WT	WT	WT	WT	P104T	Q743*
**KKU-M055**	WT	WT	R361H	WT	WT	P946Lfs*20 S949P	WT	WT	WT	WT	WT	WT	K57T	D1636Gfs*2	WT	WT	WT	WT	WT	WT	WT
**HUCC-T1**	G12D	R175H	WT	WT	WT	WT	WT	WT	WT	WT	WT	WT	WT	WT	WT	WT	WT	WT	S294*	Q2285*	WT
**HuCC-A1**	WT	G154V	WT	WT	WT	S1138*	WT	WT	WT	WT	WT	R143*	WT	WT	WT	WT	WT	WT	WT	WT	WT
**SG231**	G12F	T125P	WT	WT	WT	D2133G	Q1041*	WT	WT	WT	P3076Q	WT	WT	WT	WT	WT	C202*	WT	WT	WT	WT
**CC-LP-1**	WT	R306* P72R	WT	E590*	WT	WT	WT	WT	WT	WT	WT	WT	WT	A1670V	WT	G34V	WT	WT	WT	WT	WT

Table S2. OncoKB mutations (putative drivers and variants of uncertain significance) in HRR-related genes in cell lines overview.

	ARID1A	ATM	ATRX	BAP1	BLM	BRCA1	BRCA2	BRIP1	CHEK2	CHEK1	FANCA	FANCC	FANCD2	FANCE	FANCF	FANCG	FANCL	MRE11	NBN	PALB2	RAD50	RAD51	RAD51B	BARD1
**YSCCC**	WT	WT	WT	WT	WT	WT	WT	WT	WT	WT	WT	WT	S780L	WT	WT	WT	WT	WT	WT	WT	WT	WT	WT	S241C
**TKKK**	WT	WT	WT	WT	WT	WT	WT	WT	WT	WT	WT	WT	WT	WT	WT	WT	WT	WT	WT	WT	WT	WT	WT	WT
**RBE**	WT	WT	WT	WT	WT	WT	I1929V	WT	WT	WT	WT	WT	WT	WT	WT	WT	WT	WT	WT	WT	WT	WT	WT	WT
**OZ**	WT	WT	WT	WT	WT	WT	K2729N	N196S	WT	WT	WT	WT	WT	WT	WT	WT	WT	WT	WT	WT	WT	WT	WT	WT
**KKU-M213**	G324V	WT	WT	WT	WT	S1634C	WT	WT	WT	WT	WT	WT	WT	WT	WT	WT	WT	WT	WT	WT	WT	WT	WT	WT
**KKU-M156**	G92A	WT	WT	WT	WT	WT	WT	WT	WT	WT	WT	WT	WT	WT	WT	WT	WT	WT	WT	WT	WT	WT	WT	WT
**KKU-100**	WT	WT	WT	WT	WT	WT	WT	WT	T576A	WT	WT	WT	WT	WT	WT	WT	WT	WT	WT	WT	WT	WT	WT	WT
**KKU-M055**	P946Lfs*20 S949P	WT	WT	WT	WT	WT	WT	WT	WT	WT	A449T	WT	WT	R89L	P117T	WT	WT	WT	WT	WT	WT	WT	WT	WT
**HUCC-T1**	WT	WT	WT	WT	WT	WT	WT	WT	WT	WT	WT	WT	WT	WT	WT	WT	WT	WT	WT	WT	K722Rfs*14	WT	WT	WT
**HuCC-.A1**	S1138*	WT	WT	WT	WT	WT	WT	WT	R223C	WT	WT	WT	WT	WT	WT	WT	WT	WT	WT	WT	WT	WT	WT	WT
**SG231**	D2133G	WT	WT	WT	WT	WT	WT	WT	WT	WT	WT	WT	WT	WT	WT	WT	WT	WT	WT	WT	WT	WT	P365R	S761N
**CC-LP-1**	WT	WT	WT	C649Y	WT	WT	WT	WT	WT	WT	WT	WT	WT	WT	WT	WT	WT	WT	WT	L337S	WT	WT	WT	WT

Table S3. Splice variants in CCA cell lines detected with Illumina TSO500 panel®.

cell line	Gene	Affected Exon	Breakpoint 1	Breakpoint 2	Splice Supporting Reads	Reference Reads Transcript
**OZ**	MET	2/21	chr7:116312631	chr7:116364746	16	4481
	MET		chr7:116312992	chr7:116339124	13	3586
	MET		chr7:116364218	chr7:116364746	28	72
**TKKK**	MET	2/21	chr7:116312631	chr7:116364746	15	3735
**HuCC-A1**	MET		chr7:116381079	chr7:116381487	35	2383
**KKU-M156**	MET	2/21	chr7:116312631	chr7:116364746	10	6025
	MET		chr7:116312992	chr7:116335803	10	1090
	MET		chr7:116312992	chr7:116339124	35	4782
	MET		chr7:116340338	chr7:116345807	12	4029
	MET		chr7:116342193	chr7:116371721	10	4122
	MET		chr7:116364218	chr7:116364746	26	375
	MET		chr7:116416984	chr7:116417442	19	5400
**CC-LP-1**	MET		chr7:116381079	chr7:116381487	10	984

Table S4. *CTNNB1*/*APC* mutated CC-LP-1 + KKU-M055 vs. WT HuCC-A1 and TKKK. Kegg WNT gene set.

Probe Label	Log2 fold change	P-value	Benjamini-Yekutieli adjusted p-value
AXIN2-mRNA	10.9	1.23E-21	3.71E-18
WNT5A-mRNA	7.7	0.0000204	0.000881
GPC4-mRNA	5.37	0.000163	0.00572
PRKX-mRNA	3.08	0.00000365	0.000229
PPP3CB-mRNA	2.17	0.000802	0.0214
PRKCA-mRNA	1.71	0.00383	0.0732
PRKACA-mRNA	1.58	0.00117	0.0293
RAC3-mRNA	1.56	0.00053	0.0156
FZD7-mRNA	1.14	0.000625	0.0178
SKP1-mRNA	0.998	0.0122	0.192
RHOA-mRNA	0.946	0.00154	0.0362
CCND1-mRNA	0.709	0.00226	0.0495
FZD3-mRNA	0.612	0.02	0.292
PLCB4-mRNA	0.561	0.778	1
PPP3CC-mRNA	0.464	0.0393	0.514
RBX1-mRNA	0.458	0.00912	0.154
MYC-mRNA	0.432	0.196	1
APC-mRNA	0.293	0.516	1
CUL1-mRNA	0.118	0.756	1
CTNNB1-mRNA	0.116	0.616	1
EP300-mRNA	0.0203	0.818	1
AXIN1-mRNA	-0.0538	0.532	1
PPP3R1-mRNA	-0.102	0.705	1
GSK3B-mRNA	-0.177	0.285	1
MAPK9-mRNA	-0.226	0.295	1
MAPK8-mRNA	-0.247	0.0762	0.865
CAMK2B-mRNA	-0.296	0.434	1
SMAD4-mRNA	-0.42	0.0563	0.68
PLCB1-mRNA	-0.421	0.647	1
FZD2-mRNA	-0.454	0.546	1
TBL1XR1-mRNA	-0.499	0.00149	0.0358
WNT2B-mRNA	-0.62	0.0193	0.283
CCND3-mRNA	-0.77	0.000122	0.0045
PRKACB-mRNA	-0.821	0.132	1
RAC1-mRNA	-0.859	0.000563	0.0162
JUN-mRNA	-0.877	0.0115	0.186
TCF7L1-mRNA	-0.919	0.0142	0.222
CCND2-mRNA	-1.07	0.71	1
FOSL1-mRNA	-1.15	0.336	1
WNT3-mRNA	-1.16	0.0411	0.532
PPP3CA-mRNA	-1.23	0.00000423	0.000255
MMP7-mRNA	-1.58	0.00796	0.137
SMAD3-mRNA	-2.64	0.000000587	0.0000681
WNT7A-mRNA	-3.79	0.00000774	0.000402
TP53-mRNA	-3.84	0.0983	1
DKK1-mRNA	-4.19	0.114	1
WNT10A-mRNA	-4.52	0.00157	0.0364
RAC2-mRNA	-8.6	0.000000434	0.0000546

Table S5. Overview of genetic alterations and clinical characteristics of P1-P7.

patient identifier	**P1**		**P2**		**P3**		**P4**		**P5**		**P6**		**P7**	
(likely) pathogenic mutations	TP53	c.746G>A	FBXW7	c.1513C>T	TP53	c.817C>T	**CTNNB1**	c.133T>C	TP53	c.559+2T>C	ARID1A	c.1393C>T	BAP1	c.1182C>A
		p.R249K		p.R505C		p.R273C		p.S45P		Splicing		p.Q465*		p.Y394*
	POLE	c.242_246 delCAGTG	KRAS	c.35G>C	FBXW7	c.1513C>T			RUNX1	c.559G>A	KRAS	c.35G>T	FGFR2	c.827T>G
		p.A81fs*8		p.G12A		p.R505C				p.A187T		p.G12V		p.F276C
	**APC**	c.2879C>G	NRAS	c.182A>G	**APC**	c.3871C>T								
		p.S960*		p.Q61R		p.Q1291*								
	**APC**	c.1548G>A	PIK3CA	c.3140A>G	AXIN2	c.1571delT								
		p.K516K		p.H1047R		p.V524fs*100								
	TGFBR1	c.1007_1024 +11del	BARD1	c.1518_1519 delTGinsCA										
				p.V507M										
			**APC**	c.3340C>T										
				p.R1114*										
TMB (mut/Mb)	5.8		1.55		10.3		n.d.		3.1		1.6		8.3	
TNM	pT3, R0, G3		pT1b, L0, V0, R0, pN0, G2		pT2, R1, G2		rpT2b, L0, V0, R2, G2		pT4, pN1, cM1, R1, G2		pM1 (HEP)		ypT3, ypN0, cM1	
IPBN	yes		yes		no		no		no		unknown		no	
metachronous cancer	CRC and RCC		CRC		no		no		no		no		no	

CRC, colorectal cancer; RCC, renal cell carcinoma.

Table S6. *CTNNB1*/*APC* mutated CCA samples (P1-P4) vs. WT (P5-P7). Kegg WNT gene set.

Probe Label	Log2 fold change	P-value	Benjamini-Yekutieli adjusted p-value
WNT11-mRNA	4.43	0.0000175	0.00289
NKD1-mRNA	3.84	0.000000296	0.0000845
AXIN2-mRNA	3.61	9.93E-08	0.0000387
DKK4-mRNA	2.21	0.000222	0.0135
BAMBI-mRNA	1.9	0.0000298	0.00365
RAC3-mRNA	1.88	0.0429	0.669
PRKACG-mRNA	1.81	0.000112	0.00859
PLCB4-mRNA	1.35	0.00566	0.141
CCND1-mRNA	1.14	0.00289	0.0868
DKK2-mRNA	1.06	0.0262	0.461
FZD2-mRNA	1.01	0.0958	1
WNT10A-mRNA	0.989	0.0228	0.415
PLCB1-mRNA	0.894	0.00746	0.175
WNT2-mRNA	0.876	0.0827	1
FZD3-mRNA	0.771	0.0602	0.881
WNT16-mRNA	0.68	0.152	1
WIF1-mRNA	0.663	0.112	1
CAMK2B-mRNA	0.654	0.137	1
PPP3R2-mRNA	0.653	0.18	1
WNT10B-mRNA	0.463	0.263	1
CTNNB1-mRNA	0.416	0.263	1
WNT3-mRNA	0.406	0.313	1
AXIN1-mRNA	0.175	0.517	1
CREBBP-mRNA	0.16	0.603	1
WNT2B-mRNA	0.126	0.794	1
APC-mRNA	0.0298	0.924	1
CCND2-mRNA	-0.204	0.69	1
TCF7L1-mRNA	-0.272	0.259	1
GPC4-mRNA	-0.291	0.33	1
PPP3CC-mRNA	-0.324	0.423	1
MAPK10-mRNA	-0.346	0.389	1
MYC-mRNA	-0.356	0.488	1
CUL1-mRNA	-0.388	0.193	1
PRKCA-mRNA	-0.399	0.123	1
RHOA-mRNA	-0.448	0.0912	1
SMAD3-mRNA	-0.462	0.139	1
PPP3R1-mRNA	-0.514	0.0511	0.767
PPP3CA-mRNA	-0.527	0.207	1
FZD7-mRNA	-0.539	0.448	1
SKP1-mRNA	-0.547	0.0187	0.359

**EP300-mRNA**	-0.55	0.0561	0.827
**TP53-mRNA**	-0.553	0.0004	0.0197
**MAPK9-mRNA**	-0.617	0.0735	1
**SFRP1-mRNA**	-0.637	0.213	1
**SMAD4-mRNA**	-0.679	0.0135	0.277
**RAC1-mRNA**	-0.699	0.0046	0.121
**WNT5B-mRNA**	-0.73	0.165	1
**GSK3B-mRNA**	-0.783	0.000229	0.0136
**PRKACA-mRNA**	-0.834	0.00846	0.192
**RBX1-mRNA**	-0.841	0.000459	0.0216
**JUN-mRNA**	-0.948	0.0112	0.24
**FOSL1-mRNA**	-1.03	0.125	1
**FZD8-mRNA**	-1.14	0.0403	0.646
**TBL1XR1-mRNA**	-1.15	0.0000743	0.00664
**MAPK8-mRNA**	-1.32	0.000645	0.0281
**PPP3CB-mRNA**	-1.33	0.000661	0.0281
**CCND3-mRNA**	-1.36	0.000292	0.0161
**WNT5A-mRNA**	-1.65	0.00209	0.07
**PRKX-mRNA**	-1.94	0.000308	0.0167
**SFRP4-mRNA**	-2.1	0.00338	0.0959
**RAC2-mRNA**	-2.18	0.0000971	0.00771
**MMP7-mRNA**	-2.57	0.000312	0.0167
**PRKACB-mRNA**	-2.73	0.000383	0.0191
**LEF1-mRNA**	-2.91	0.000517	0.0236
**SFRP2-mRNA**	-3.63	0.000000157	0.000056
**DKK1-mRNA**	-3.66	2.14E-10	0.000000184
